# Evaluating the Reliability and Validity of the Children’s Vitality-Relaxation Scale

**DOI:** 10.3390/ijerph16183369

**Published:** 2019-09-12

**Authors:** Kyung-Sook Bang, Sungjae Kim, Kalevi M. Korpela, Min Kyung Song, Gumhee Lee, Yeseul Jeong

**Affiliations:** 1 Faculty of College of Nursing, The Research Institute of Nursing Science, Seoul National University, Seoul 03080, Korea; ksbang@snu.ac.kr (K.-S.B.); sungjae@snu.ac.kr (S.K.); 2 Faculty of Social Sciences/Psychology, Tampere University, FIN-33014 Tampere, Finland; kalevi.korpela@tuni.fi; 3 College of Nursing, Seoul National University, Seoul 03080, Korea; lghpw@snu.ac.kr (G.L.); yeaseul.jeong@gmail.com (Y.J.)

**Keywords:** affect, child, scale development

## Abstract

This study developed the Children’s Vitality-Relaxation Scale (CVRS) by revising the adult version of the Restoration Outcome Scale (ROS). The CVRS was translated and culturally adapted into Korean, and its reliability and validity were evaluated in a cross-sectional, descriptive design study. Data collected from 181 elementary school students in grades 4‒6 were used to test the validity and reliability of the CVRS. Exploratory factor analysis, Pearson’s correlation, known-groups comparison, and Cronbach’s alpha were used for analysis. The factor analysis indicated a two-factor structure, and all factor loadings were above 0.40. The CVRS was a seven-point Likert scale consisting of eight items, which were classified as “vitality” (four items) and “relaxation” (four items). The external construct validity with the PANAS, PSS, and SRI was acceptable. In the known-groups comparison, the CVRS score was significantly higher for boys than for girls, and the CVRS score for high-income students was higher than low-income students. The Cronbach’s α for the scale was 0.84 and ranged from 0.72–0.87 for the subscales. Results showed that the CVRS is a valid and reliable scale with acceptable psychometric characteristics in Korean children. The scale can be used to measure children’s affect in various settings.

## 1. Introduction

Affect is “a neurophysiological state that is consciously accessible as a simple, non-reflective feeling (e.g., of pleasure or displeasure)” [1] (p. 147) and is related to an individual’s physiological, psychological, and social health. Also affect is known to influence health behaviors [2]. In modern competitive society, mental health problems and stress in people are worsening, and children are no exception to this. 

Negative affect (NA) is defined as the experience of emotional distress or unpleasantness experienced by an individual [3,4]. NA is experienced as unpleasant, unwanted emotions and includes negative emotions and feelings such as negativity, badness, anger, sadness (depression) and/or fear (anxiety) [5]. Negative affect (NA) influences the physical, psychological, and social health of children [6,7,8,9], affecting not only childhood health, but also one’s lifetime health status [10,11]. Studies have primarily focused on the effects of NA on children’s health, development, and relationships [6,7,8,9]; outcome evaluations of intervention programs to decrease NA [12,13]; and the development of suitable tools to detect NA [14,15,16,17].

Positive affect (PA) is defined as a pleasant or positive emotional experience [18]. It includes both positive emotions and feelings, and consists of positive, good, pleasant, happy, and satisfying feelings or experiences [5]. Although there is sufficient scientific evidence of the effect of PA on health in adults and children [19,20,21], there has been little research interest in developing an instrument to measure this effect. Positive affect is generally assessed using the Positive Affect and Negative Affect Schedule (PANAS) or Profile of Mood States (POMS) in all populations, which are reliable and valid tools to measure affect [22,23], and are used to evaluate outcomes of intervention programs and to examine children’s affect in the field of academics.

Russell [1] explained affect by categorizing it into two dimensions. One is the hedonic dimension of pleasure–displeasure, and the other is the arousal dimension of calm–activated. According to Russell, affect can exist in the positive–negative as well as the arousal–nonarousal dimensions. For example, if placed on a grid, excitement and contentment, which are PA, are placed in different quadrants of the grid depending on the levels of arousal or nonarousal. Distress and depression, which are NA, are also classified based on the arousal status [24]. In other words, the measures that evaluate affect should identify the affect accurately at both positive–negative and arousal–nonarousal levels. The POMS was developed to determine the state (tension, anxiety, anger, depression, vigor, and fatigue) of patients with a psychiatric disorder, but it has limitations in measuring other affects. The PANAS can be used to measure high arousal states in PA and NA; however, it is not suitable for evaluating nonarousal states such as serenity and fatigue [25], and this limitation was reported in a previous study [26].

PA in childhood is related to the formation of a stable attachment with parents during infancy, and in later life, it might be related to a positive close relationship [27]. As PA in childhood has a lifetime influence on health status, it is necessary to develop a program that can improve PA, and to construct a suitable and reliable scale to evaluate intervention outcomes. In other words, it is necessary to develop a simple tool to measure the arousal–nonarousal PA of a child that allows children to easily understand and respond to the questions.

PA based on the arousal dimension has been traditionally measured in adults with the affect grid and/or the Restoration Outcome Scale (ROS). The affect grid was first published in 1989 as a single-item measure for two dimensions of influence: pleasure-displeasure and calm-activated [28]. The affect grid is moderately valid measure of the dimension of general pleasure and arousal, but it has been reported that there is little specificity in distinguishing the qualities of various affective experiences [29]. Korpela, Ylén, Tyrväinen, and Silvennoinen [30] developed the ROS based on the attention restoration theory and previous measures [31,32,33] to measure restoration outcome in adults after exposure to nature. ROS is based on the theory that exposure to nature helps individuals relax, increases PA, eliminates unwanted thoughts, and improves attention and vitality [34]. The ROS comprises items which cover relaxation and calmness, attention restoration, clearing one’s thoughts, subjective vitality, and self-confidence. The ROS can measure arousal–nonarousal PA at present [35] 

In this study, we revised the adult version of the ROS and developed the Children’s Vitality-Relaxation Scale (CVRS) using simple vocabulary [36] for children. The scale was then translated into Korean. The reliability and validity of the Korean version of the CVRS to measure children’s affect was evaluated.

## 2. Materials and Methods 

### 2.1. Participants

Participants were elementary school students in grades four to six (ages 10–12) who agreed to participate in the study. For exploratory factor analysis to test a scale’s validity, the required sample size should be at least 5–20 times the number of items in the study [37]. Therefore, the required sample size in this study was 180. Considering an incomplete response rate of 10%, the final sample consisted of 200 participants. To increase the validity of the scale, community child centers were also included as research sites to include vulnerable children with difficult family conditions and/or children from low-income families who were more likely to be exposed to psychosocial health risk factors. The participating institutions were selected using convenience sampling. Three elementary schools in Seoul and Gyeonggi-do and three community children centers in Seoul agreed to cooperate after the purpose of the study was explained. Participants in the survey attended selected institutions, and the exclusion criteria were as follows: students under 10 years old, students who have difficulty reading and writing Korean, and students whose parents did not give consent to participate in the study. Of the 259 eligible participants, 200 completed the questionnaire. After excluding questionnaires with incomplete responses, data from 181 questionnaires were analyzed.

### 2.2. Translation and Cultural Adaptation Processes

The adult version of the ROS was modified for children by collaborating with the original developer of the scale. The revised scale was independently translated from English to Korean by two researchers based on the Brislin translation model [38,39]. The research team reviewed the items of both Korean-translated versions of the scale. The two versions were then integrated into one (first Korean version), considering the vocabulary and syntax of the questions, age characteristics of the children, and cultural meaning of the items. The integrated tool was back translated into English by a person fluent in English and Korean. The back translated tool was then translated back into Korean by a simultaneous interpreter (final Korean version). Finally, five researchers compared the translated Korean version of the scale and the first Korean version of the scale to determine whether the translation was accurate without any change in meaning and approved the final Korean version of the scale. No items were excluded during the translation process.

### 2.3. Instruments

Instruments used for children in previous studies were selected, and items’ suitability for measuring children’s affect was reviewed by two experts. A preliminary survey was also conducted on children of the same age to assess the suitability of the scales.

#### 2.3.1. The Children’s Vitality-Relaxation Scale (CVRS)

Based on the ROS, we developed a scale that could evaluate children’s state of PA and measure change in PA as the result of an intervention. Each item was modified using simpler words to help children understand. The ROS scale comprises nine items, which are rated on a seven-point Likert scale (where 1 = “not at all” and 7 = “completely”). Higher scores indicate better outcomes. In order to compare the changes in children’s affect, the word “here” was deleted from the original items of the ROS, and each item of the CVRS was revised to the present tense. At the time of scale development, the Cronbach’s alpha was 0.92 [40]. Cronbach’s α of the subscales ranged from 0.72 to 0.87, and in this study, the Cronbach’s α of the total scale was 0.84.

#### 2.3.2. Positive Affect and Negative Affect Schedule (PANAS)

The PANAS, developed by Watson, Clark, and Tellegen [4] and translated into Korean by Lee, Kim, and Lee [41], was used to measure a child’s emotional state and to assess the external construct validity of the questionnaire based on its correlation with the CVRS. The scale consists of 10 PA and 10 NA words, which are rated on a five-point Likert scale ranging from 1 (“not at all”) to 5 (“extremely”). Higher scores indicate higher affect. Lee et al. [41] reported high Cronbach’s alphas for both PA (0.88) and NA (0.85); in the present study, the Cronbach’s alphas for PA and NA were 0.86 and 0.84, respectively.

#### 2.3.3. Perceived Stress Scale (PSS)

The PSS, developed by Cohen et al. [42] and translated and modified into Korean by Lee [43], was also used to evaluate the external construct validity of the CVRS. This 10-item instrument measures the degree to which everyday events are perceived as stressful for adolescents in Korea. Each item is rated on a four-point Likert scale from 0 (“never”) to 4 (“very often”), with the total score ranging from 0 to 40. The negative items are reverse scored, and higher scores indicate a higher level of perceived stress. The Cronbach’s alpha was 0.76 in a study on adolescents [44], and in the present study, the Cronbach’s alpha was 0.81. 

#### 2.3.4. Stress Response Inventory (SRI)

The Stress Response Inventory (SRI) designed to measure stress levels in children was used to assess the external construct validity of the CVRS. This study used the 22-item modified stress response inventory (SRI-MF) derived from the original SRI questionnaire [45]. The instrument consists of 22 questions including nine items on somatization, eight on depressive symptoms, and five on anger. Each item is rated on a five-point Likert scale from 0 (“not at all”) to 4 (“very much”). At the time of scale development, the Cronbach’s alpha was 0.93; in this study, the Cronbach’s alpha was 0.94.

### 2.4. Data Collection

The grades to be included in this study were selected after discussion with the principals and homeroom teachers. The researchers distributed the consent form to elementary and community child center students. The consent form included information about the purpose and method of the study, anonymity of the participants, and the ability to withdraw consent. Written consent was requested from both the legal guardians and students. After explaining how to respond to the questionnaire, questionnaires were distributed to consenting participants. It took about 20 minutes to complete the questionnaire, and a small gift was provided to students upon completion. Data were collected from 8 October 2018 to 30 October 2018.

### 2.5. Data Analyses

Data were analyzed to determine the reliability and validity of the CVRS using SPSS WIN 21.0 (IBM, Armonk, NY, USA) and LISREL 8.80 (Scientific Software International, Lincolnwood, IL, USA, 2006). The general characteristics of the participants are presented as descriptive statistics. The validity of the scale was evaluated by testing for construct validity. An exploratory factor analysis using a polychoric correlation matrix was performed to analyze the construct validity of the CVRS. The Kaiser–Meyer–Olkin (KMO) and Bartlett’s tests were used to evaluate whether the sample was adequate to perform a satisfactory factor analysis. A known-groups comparison was performed to assess how CVRS scores differed among participants’ demographic subgroups, and an independent t-test, ANOVA, Mann–Whitney test, and Kruskal–Wallis test were performed. Pearson’s correlation was used to assess the external construct validity. The reliability of the scale was calculated in terms of internal consistency by computing Cronbach’s alpha coefficient. In addition, Cronbach’s alpha coefficients were computed to determine the reliability of each factor. 

### 2.6. Ethical Approval

This study was conducted in accordance with the Declaration of Helsinki, and ethical approval was obtained from the Seoul National University Ethical Review Board (IRB No. 1809/002-007), after which data were collected and analyzed. Only students and their legal guardians who provided written consent were included in this study. The school boards of six elementary schools agreed to provide children and their legal guardians with all the necessary information and the informed consent forms. In addition, participation in the research was voluntary.

## 3. Results

### 3.1. Characteristics of the Participants and Descriptive Data

Table 1 presents participant characteristics, including the type of institution, gender, grade, family structure, perceived economic status, and academic level. A total of 181 children from three community centers for children (*n* = 34, 18.8%) and three regular elementary schools (*n* = 147, 81.2%) were included in the study. There were 84 girls (46.4%) and 97 boys (53.6%). The participants included 39 (21.5%) fourth grade, 77 (42.5%) fifth grade, and 65 (35.9%) sixth grade children, with a mean age of 11.14 ± 0.75 years. Most of the children (90.1%) were living with their parents. Regarding perceived economic status, 99 (54.7%) children stated that their economic status was high, and 82 (45.3%) children stated they were from middle- and low-income families. In terms of academic level, 97 (53.6%) children considered themselves to be high achievers, 68 (37.6%) responded that they were moderate achievers, and 16 (8.8%) responded that they were low achievers (Table 1). 

### 3.2. Construct Validity 

In order to test the construct validity of the CVRS used in this study, exploratory factor analysis was performed on nine items. The construct validity of this study was confirmed through factor analysis and the known-groups validity method.

#### 3.2.1. Factor Analysis 

**Figure 1:**
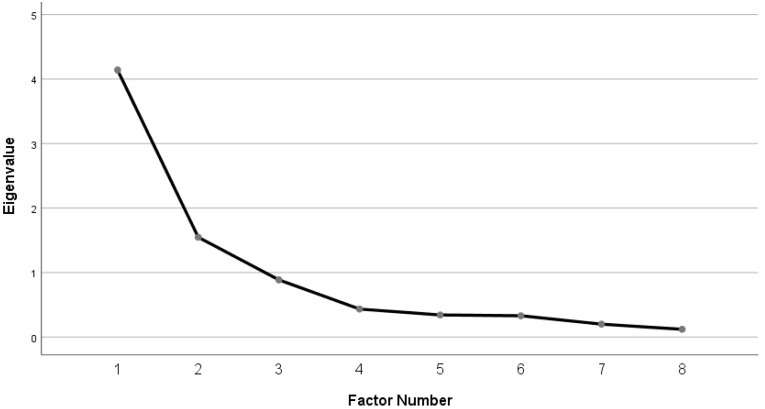
Scree plot for factor components of the Child Vitality-Relaxation Scale.

In order to confirm the appropriateness of the exploratory factor analysis, we conducted the sphere formation test of KMO and Bartlett’s test of sphericity. The KMO measure of sampling adequacy for the nine items was 0.761, and Bartlett’s test of sphericity, which indicates the suitability of the factor analysis model, was significant (χ² = 884.0; *p* < 0.001), indicating that the correlation coefficient matrix was suitable for factor analysis. The collected data were analyzed by principle component analysis using Varimax Rotation to minimize the number of factors and information loss. The factor loadings of all items were found to be over 0.40. The range of eigenvalues in the data ranged from 4.14 to 0.12. Using the criterion of eigenvalue > 1, two factors were extracted. The scree plot showed that the CVRS consisted of two factors (Figure 1).

The first factor included five items related to the vitality, confidence, and energy of the children, and it was defined as “Vitality.” Of these, item 6, “My thoughts are clear” showed loading values of 0.4 or higher on factors 1 and 2. Item 6 was deleted on the basis that if the loading value of a specific item appears to be similar for several factors it is judged that it does not belong to any factor [46]. The first factor had an eigenvalue of 4.14 and accounted for 36.3% of the variance in data. The second factor included four items related to calmness, relaxation, and having no worries, and it was defined as “Relaxation”. It had an eigenvalue of 1.55 and accounted for 25.5% of the variance in data. The communality range for all of the final eight items was 0.40 to 0.85, and the factor loadings ranged from 0.55 to 0.91. The factor loadings for all items are shown in Table 2. 

#### 3.2.2. Known-Groups Validity

In order to assess whether the CVRS sensitively measures children’s vitality and relaxation levels, we compared the degree of vitality-relaxation and children’s general characteristics. The analysis showed that the level of relaxation in children from the community child center was significantly lower than that of children from the schools (*p* = 0.023), and the CVRS scores of girls were lower than those of boys (*p* = 0.010). As the grade increased, children’s CVRS scores decreased (*p* = 0.009). As grade increased, the level of vitality decreased (*p* = 0.002). Cohabitation with parents did not have an impact on the CVRS scores of the children (*p* = 0.194). The children with lower perceived economic and academic status were found to have lower CVRS, vitality, and relaxation scores (*p* < 0.001) (Table 1). 

#### 3.2.3. External construct Validity

The external construct validity was determined by analyzing the correlations of the CVRS with the PANAS, PSS, and SRI using Pearson’s correlation coefficient. The overall CVRS was positively correlated with PANAS positive (*r* = 0.543, *p* < 0.001), and it was negatively correlated with PANAS negative (r = −0.337, *p* < 0.001), PSS (r = −0.583, *p* < 0.001), and SRI (r = −0.448, *p* < 0.001) (Table 3). 

### 3.3. Reliability 

The Cronbach’s alpha coefficient of the CVRS was 0.84, indicating a high internal consistency. Cronbach’s alpha coefficient was 0.87 for the first factor and 0.72 for the second factor (Table 2). 

## 4. Discussion

The purpose of this study was to develop a scale to assess PA in children based on the adult version of the ROS and to evaluate its validity and reliability. This study showed that the CVRS is a sound and reliable tool that can be used by researchers.

The exploratory factor analysis revealed a two-factor structure that accounted for 61.8% of the variance, unlike the original ROS, which had a single-factor structure. Factor 1 was loaded with items related to subjective vitality (“I feel lively and vital”, “I am hopeful”), self-confidence (“I feel self-confident”), and feeling energetic (“I am enthusiastic and energetic”), which were included in the “relaxation” factor of the ROS. Factor 1 was defined as “vitality” because the items reflected subjective vitality, defined by Ryan and Frederick [47] as a subjective experience full of energy and survival. Vitality reflects PA associated with a higher level of awakening compared to a state of calmness [48]. Increasing vitality refers to increasing energy and mental agility, and decreasing vitality refers to tiredness [49]. Factor 2 was loaded with items related to relaxation and calmness (“I feel calm”, “I feel relaxed”), concentration (“I can concentrate well”), and clearing one’s thoughts (“I do not have any worries”). Factor 2 was defined as “relaxation” because the items covered relaxation, concentration, and not having worries. The two factors included ranges from not at all aroused (e.g., relaxed, calm) to aroused (e.g., stimulated, vitality) [50]. The final CVRS consisted of eight items after deleting one item (“My thoughts are clear”), which cross-loaded on multiple factors.

The validity of the CVRS was examined using the known-groups comparison. First, the relaxation level of children from the local community child center was significantly lower than that of children from regular elementary schools (*p* = 0.023). In South Korea, community child centers are primarily used by children from vulnerable social groups such as single-parent households, living with grandparents, multicultural children, and low-income families [51]. Our study suggests that the socioeconomic status of the family has an effect on the child’s affect, and these findings are consistent with those of Gellci et al. [52]. 

In terms of gender, boys’ vitality was higher than that of girls. This indicates a difference in the level of PA depending on gender, similar to the findings of Moon and Cho [53], who found a high level of NA in girls when compared to boys. 

With respect to grade, vitality was lower in children from higher grades. In addition, among the general characteristics, perceived academic status and perceived economic status had the greatest impact on children’s vitality and relaxation. In South Korea, where academic success and achievement are prioritized [54], elementary school children experience psychological pressure due to the emphasis on academic achievement, especially in the senior year [54]. These findings reflect the reality in South Korea, where academic achievement of children is emphasized.

Since the original scale was developed for adults, it is difficult to directly compare the characteristics of the participants. However, in this study, the construct validity of the CVRS was confirmed by known-groups comparison. The results of this study suggest that the CVRS is an excellent instrument that can sensitively measure the level of vitality and relaxation in children. 

The Cronbach’s alpha coefficients of all the items in the CVRS were as high as 0.84, and the Cronbach’s alpha coefficients of the subscales were more than 0.70. A coefficient level higher than 0.70 [55] indicates that the scale is reliable.

To confirm the external construct validity, this study analyzed the correlations of the CVRS with the PANAS, PSS, and SRI. There was a positive, significant correlation between the CVRS and PA on the PANAS, and a significant negative correlation between the CVRS and NA on the PANAS. There were strong correlations between vitality (one of the subscales of the CVRS) and PA on the PANAS and between relaxation and NA on the PANAS. A high PA indicates high levels of energy, concentration, and pleasure [4]. Vitality is the sense of being fully energetic and alive from within [47] and is classified as arousal—PA, which is associated with high PA on the PANAS. Meanwhile, relaxation is negatively correlated with NA on the PANAS. Low NA is interpreted as a calm and serene state [4], which is a nonaroused PA. As low PA does not indicate a nonaroused positive state in the PANAS, PA and NA should be assessed together in order to evaluate the overall PA of participants. Unlike the PANAS, the CVRS can measure both arousal and nonarousal degrees of PA using only eight items.

In this study, PA measured using the CVRS was negatively correlated with PSS and SRI, and this finding is in accordance with that of a previous study by Lindeberg et al. [56] that reported the correlation between low vitality (exhaustion) and chronic stress (reduced activity in the hypothalamo-pituitary-adrenal axis). Furthermore, a stressful stimulus activates the sympathetic nervous system. The autonomic nervous system response leads a relaxed individual into an alert state and prepares them to confront the stimulus. This basic physiological change might explain the negative correlations of relaxation with PSS and SRI.

This study has some limitations. First, although we tried to include participants from different settings, all participants were recruited from the metropolitan areas of Korea. In order to make the findings of this study more generalizable, it is necessary to include participants from more regions and countries. Second, this study used the Korean version of the CVRS. Although the research team rigorously followed the translation guidelines and cultural adaptation processes, the English version of the CVSR has not been tested and further research should be conducted to do so.

It is important to use tools that are appropriate for evaluating PA in arousal and nonarousal states in order to adequately measure the effects of interventions aimed at mitigating stress and improving PA in children. The CVRS is composed of simple items that are easy for children to understand and can be used to assess the affective state in arousal–nonarousal states in children at the time of measurement. The CVRS also can be used as a tool to measure an outcome variable to assess the effectiveness of interventional programs to improve children’s mental health and PA, such as forest-based activities or play therapy.

## 5. Conclusions

PA is an important concept for school-aged children, and suitable instruments are needed to objectively evaluate PA in children. In this study, we developed a tool to simply and quickly measure vitality and relaxation in children as part of the PA. The CVRS is a self-report eight-item, seven-point Likert scale consisting of two subscales—vitality and relaxation. The study found that the level of relaxation of children in the community child centers was lower than that of children in general elementary schools, the level of vitality and relaxation of girls was lower than boys, and the level of vitality decreased in children in higher grades. In addition, the validity of the scale was verified by confirming that both vitality and relaxation were lower when perceived economic status and academic levels were lower. The external construct validity was confirmed by the results of the moderate correlation of the CVRS with the PANAS, PSS, and SRI. 

The CVRS measures vitality and relaxation in Korean children easily when compared to observational tools, thereby making it more cost- and time-effective, and more applicable in diverse settings.

## Figures and Tables

**Table 1:** General characteristics of the participants and score of CVRS (*n* = 181).

Variables	Categories	*n* (%)	CVRS		Vitality		Relaxation	
			M (SD)	t/F	M (SD)	Z/H	M (SD)	Z/H
				(*p*)		(*p*)		(*p*)
Type of institution	School	147 (81.2)	38.44 (8.79)	1.64 (0.102)	21.05 (5.40)	−0.64 (0.523)	17.39 (4.80)	−2.27 (0.023)
	Community center	34 (18.8)	35.65 (9.43)		20.32 (5.99)		15.32 (4.86)	
	Sub-total		37.91 (8.95)		20.91 (5.51)		17.00 (4.87)	
Gender	Female	84 (46.4)	36.08 (9.17)	2.60 (0.010)	19.82 (5.63)	−2.53 (0.011)	16.26 (5.09)	−2.21 (0.027)
	Male	97 (53.6)	39.49 (8.50)		21.86 (5.24)		17.64 (4.60)	
	Sub-total		37.91 (8.95)		20.91 (5.51)		17.00 (4.87)	
Grade	4th	39 (21.5)	41.79 (7.32)	4.88 (0.009)	23.64 (4.24)	12.82 (0.002)	18.15 (4.73)	4.01 (0.135)
	5th	77 (42.5)	36.92 (9.46)		20.49 (5.71)		16.43 (5.21)	
	6th	65 (35.9)	36.75 (8.70)		19.77 (5.50)		16.98 (4.46)	
	Sub-total		37.91 (8.95)		20.91 (5.51)		17.00 (4.87)	
Family structure	With parent	163 (90.1)	38.21 (8.87)	−1.30 (0.194)	21.07 (5.40)	−1.08 (0.279)	17.13 (4.89)	−1.02 (0.308)
	Without both parents	18 (9.9)	35.22 (9.53)		19.44 (6.40)		15.78 (4.60)	
	Sub-total		37.91 (8.95)		20.91 (5.51)		17.00 (4.87)	
Perceived economic status	High	99 (54.7)	40.10 (9.00)	−3.74 (<0.001)	22.35 (5.25)	−4.04 (<0.001)	17.75 (5.17)	−2.28 (0.023)
	Middle and low	82 (45.3)	35.27 (8.20)		19.17 (5.34)		16.10 (4.33)	
	Sub-total		37.91 (8.95)		20.91 (5.51)		17.00 (4.87)	
Perceived academic status	High	97 (53.6)	41.34 (7.69)	19.63 (<0.001)	22.65 (4.65)	20.01 (<0.001)	18.69 (4.54)	27.28 (<0.001)
	Moderate	68 (37.6)	34.60 (8.51)		18.99 (5.61)		15.62 (4.29)	
	Low	16 (8.8)	31.19 (9.27)		18.56 (6.53)		12.62 (4.77)	
	Sub-total		37.91 (8.95)		20.91 (5.51)		17.00 (4.87)	

CVRS: Child Vitality-Relaxation Scale.

**Table 2:** Factor analysis and reliability of the Child Vitality-Relaxation Scale.

Factor		Items	Communality	Factor Loading		M (SD)	Corrected Item-Total Correlation	Cronbach’s α If Item Deleted	Cronbach’s α
				F1	F2				
F1 Vitality	3	I am enthusiastic and energetic.	0.79	0.88		5.51 (1.49)	0.75	0.821	0.867
	7	I feel confident.	0.66	0.76		4.93 (1.72)	0.73	0.823	
	8	I feel lively and vital.	0.85	0.91		5.42 (1.64)	0.79	0.801	
	9	I am hopeful.	0.69	0.61		5.06 (1.66)	0.62	0.871	
F2 Relaxa-tion	1	I feel calm.	0.43		0.64	3.74 (1.54)	0.42	0.700	0.715
	2	I can concentrate well.	0.40		0.55	4.91 (1.47)	0.47	0.675	
	4	I am relaxed.	0.72		0.74	4.66 (1.69)	0.62	0.576	
	5	I have no worries.	0.44		0.60	3.69 (1.90)	0.52	0.648	
	Eigenvalue			4.14	1.55				
	Variance (%)			36.3	25.5				
	Cumulative variance (%)			36.3	61.8				
Cronbach’s α for the total CVRS = 0.835									

Extraction method: principal component analysis, Rotation method: varimax with Kaiser normalization. M: mean; SD: standard deviation.

**Table 3:** Correlations among the CVRS, PANAS, PSS, and SRI.

Variables		CVRS		
		Total	Vitality	Relaxation
		r (*p*)	r (*p*)	r (*p*)
Vitality		0.880 (<0.001)		
Relaxation		0.844 (<0.001)	0.488 (<0.001)	
PANAS	Positive	0.543 (<0.001)	0.624 (<0.001)	0.293 (<0.001)
	Negative	−0.337 (<0.001)	−0.187 (0.012)	−0.408 (<0.001)
PSS		−0.583 (<0.001)	−0.431 (<0.001)	−0.584 (<0.001)
SRI		−0.448 (<0.001)	−0.298 (<0.001)	−0.488 (<0.001)

CVRS: child vitality-relaxation scale; PANAS: positive affect and negative affect schedule; PSS: perceived stress scale; SRI: stress response inventory.
